# T-cell subset changes during the first year of pre-seasonal allergoid allergen-specific immunotherapy

**DOI:** 10.1016/j.heliyon.2023.e21878

**Published:** 2023-11-10

**Authors:** Manuel Reithofer, Simone Lisa Boell, Claudia Kitzmueller, Fritz Horak, Barbara Bohle, Beatrice Jahn-Schmid

**Affiliations:** a Department of Pathophysiology and Allergy Research, Medical University of Vienna, Vienna, Austria; b Institute of Molecular Biotechnology, University of Natural Resources and Life Sciences, Vienna, Austria; c Allergiezentrum Wien West, Vienna, Austria

## Abstract

Allergen-specific immunotherapy (AIT) is the only treatment for type I allergy, which achieves long-lasting effects. Repeated subcutaneous applications of allergen extracts cause a protective antibody response and an immune deviation of T cells. In AIT with allergoids, chemically modified allergen extracts are injected. During a so-called special pre-seasonal application scheme, after the initial phase of applying increased doses of allergoids is followed by natural allergen exposure as a maintenance phase. The effectiveness of allergoid vaccines has been described regarding the improvement of clinical symptoms and the development of protective humoral responses.

In this longitudinal observational study, we sought to investigate changes at the T cell level in pre-seasonal AIT with allergoid. Different subsets within CD4^+^ and CD8^+^ T cells were monitored by flow cytometry in PBMC of patients known to possess protective antibody responses.

Compared to before treatment, a small early boost among allergenic Th cells was observed after 4 months of AIT. In line, a slight Th2 bias was observed after 4 months within circulating T follicular T cells, Tfh and Tfc, representing pre-existing memory Th2 cells. Furthermore, it was demonstrated that responsiveness of CD8^+^ T cells to allergen stimulation decreased during the course of treatment. Apart from that, we found an influence of the meteorological season on the activation profile of Tfh and Tfc over the course of the treatment.

Together, this is the first study investigating changes of different T cell subsets over the course of an allergoid AIT against airborne allergens. Our findings match previous reports on conventional AIT, especially the initial increase of Th2 responses. However, the observed changes were less pronounced which may be either due to the modification of allergens or to the reduced maintenance dose provided by natural allergen exposure compared to a perennial protocol.

## 1 Introduction

Currently, the only causative treatment of IgE-mediated allergy is allergen immunotherapy (AIT). Conventional subcutaneous AIT (scAIT) comprises multiple injections of either native allergen extracts or chemically modified extracts (allergoids) with reduced risk for adverse events for at least 3 years [1,2]. In a pre-seasonal regimen, allergic patients receive increasing doses of the vaccine until reaching the maintenance phase. The natural uptake of allergen during the season substitutes for the administration of maintenance dose. Between the seasons patients receive regular vaccinations containing maintenance doses. It is well established that conventional AIT induces changes in allergen-specific humoral and cellular immune responses, such as induction of specific immunoglobulin (Ig) G4 antibodies with IgE-blocking activity, a shift from IL-4 dominated allergen-specific Th2 response towards a Th1/0 response with increased interferon-gamma (IFN-g) production, as well as an increase in suppressive regulatory T (Treg) cells [[3], [4], [5]]. In secondary lymphoid organs follicular CXCR5^+^CD4^+^ Th cells (Tfh) characterized by the expression of the transcription factor Bcl6 have been shown to promote B cell proliferation, immunoglobulin (Ig) hypermutation and consequently the generation of memory B cells and plasma cells producing high affinity antibodies [[6], [7], [8], [9]]. In analogy to classical CD4^+^ Th cells, defined by the production of the signature cytokines IFN-g, IL-4 and IL-13, or IL-17, Tfh also include functional subsets called Tfh1, Tfh2, and Tfh17 cells separated by similar cytokine profiles and transcription factors [10]. Circulating CXCR5^+^ Tfh (cTfh), which represent recent immunological events in B cell follicles, can be found in peripheral blood, for example, virus-specific, functional cTfh with antibody-promoting activity have been detected in subjects vaccinated with viral vaccines [[11], [12], [13], [14], [15]]. Moreover, Tfh2 play an important role in the onset of allergic diseases by producing IL-4 and IL-13 [16,17]. Concordantly, an increased polarization of CXCR5^+^ T cells towards the Tfh2 lineage has been associated with allergic asthma [[18], [19], [20]]. Furthermore, a significant reduction of the CXCR5^+^ cTfh population in the course of grass-pollen scAIT has been described [21]. More recently, during a short-course AIT with hydrolysates from *Lolium perenne* pollen extract, a drastic reduction of IL-4^+^ Tfh and an increase in IFN-g^+^ Tfh accompanied by an increase of Tfreg cells has been reported [22,23]. Notably, changes in Tfh cell populations were positively correlated with the clinical improvement in these patients [24].

Besides CD4^+^ Tfh, also CD8^+^ T cells expressing CXCR5, called Tfc, have been described [25]. Different subsets of these cells exist and can have cytotoxic function or either impair or support antibody responses [26]. In persistent chronic viral infections, such as Epstein-Barr or human immunodeficiency virus infections, that these cytotoxic CXCR5^+^ CD8^+^ T cells govern control mechanisms over infected T(fh) and B cells [25,27,28]. However, the role of this cell type in allergy as well as AIT is so far not well characterized. Yet, they could display an interesting target of AIT as it was previously indicated that Tfc have the ability to induce antibody production in B cells after functional interaction and via IL-21 signaling [29,30].

Apart from follicular T cells, allergen-specific highly differentiated CD27^-^Th2 CD4^+^ cells expressing the chemoattractant receptor-homologous molecule (CRTh2) have been associated with allergic disease [31]. These “allergenic Th2” (aTh2) cells have also been found to be preferentially deleted during alder pollen AIT whereas the CD27^+^Th2 subset persists during the course of treatment [31]. An earlier study in individuals undergoing AIT with peptides derived from the major cat allergen Fel d 1 showed an significant reduction of this terminally differentiated population when compared to placebo-treated individuals [32]. CRTh2 can also be expressed on CD8^+^ Tc2 cells [33]. While the presence of CD8^+^ T cells expressing high levels of IL-4 has been described early on in patients with mild atopic asthma, expression of CRTh2 on these cells has not been analyzed [34].

In this longitudinal observational study, we used PBMC from grass pollen-allergic patients who were treated with grass-pollen allergoids in a pre-seasonal regimen and had developed protective antigen-specific humoral responses. As T cells direct and modulate humoral immune responses we sought to investigate several rarely studied CD4^+^ or CD8^+^ T cell sub-populations to identify their potential involvement in cellular immune mechanisms operative during scAIT.

## 2 Methods

### 2.1 Study population and sample preparation

**Fig. 1:**
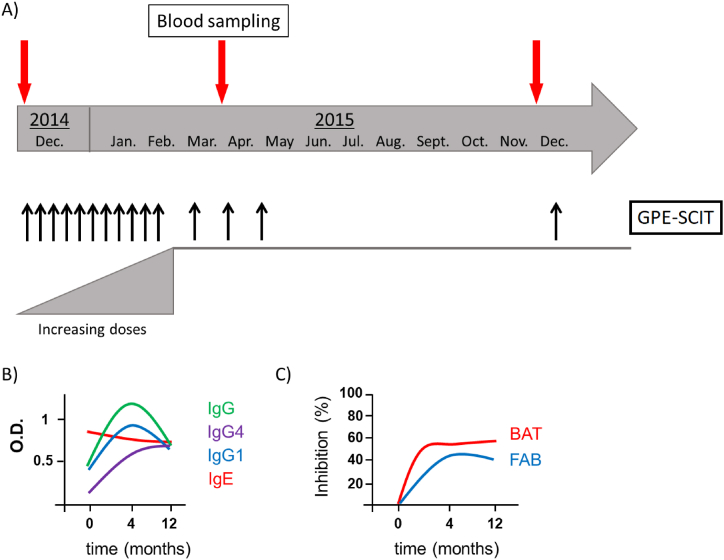
**Study design and humoral response during SCIT.** A. Treatment and blood sampling schedule of SCIT with grass pollen (GP) allergoid. B, C. Schematic graphs representing previously published data regarding GP-specific antibody responses. B. GP-specific IgE and IgG. C. Inhibiton of basophil activation and facilitated antigen binding by patients' sera.

The study included ten patients aged 19–43 years (median age 31 years, 7 male, 3 female) treated with Allergovit® Grass (Allergopharma GmbH & Co KG, Germany) in a pre-seasonal regimen for 1 year as outlined in Fig. 1A. The vaccine contained an allergoid consisting of pollen extracts of 6 grass species with about 25 μg of Phl p 5 or homologous allergens, respectively, per injection of peak and maintenance doses [35]. Heparinized peripheral blood was obtained at the indicated time points (Fig. 1A) and PBMC were isolated by Ficoll-Paque (Pharmacia Diagnostics, Uppsala, Sweden) gradient centrifugation and cryo-conserved in liquid nitrogen. The study was approved by the local ethics committees (No. EK-05/14 and No. 1593/2016) and all patients gave written informed consent.

### 2.2 Flow cytometry

PBMC from the same individual isolated at different time points and analyzed within the same experiment to minimize experimental deviations. After thawing, the cells were rested for 16 h at 37 °C in RPMI 1640 (Sigma Aldrich, Darmstadt, Germany) supplemented with 10 % autologous serum. After incubation for 20 min at 4 °C with 20 % human AB-serum in PBS cells were stained for 30 min with the following fluorophore-labelled antibodies at 4 °C: CD3-BV510 (UCHT1), CD27-PE/Cy7 (O323), CD45RA-FITC (HI100), CD278-APC/eFlour 780 (ISA-3), CD279-APC (J105), CXCR5-PE (MU5UBEE), CRTh2-APC (BM16) (BD Biosciences, San Jose, CA, USA), CD4-PE/Cy7 (SK3), CD56-FITC (TULY56), CXCR3-PerCP/Cy5.5 (G025H7), CCR6-BV421 (29-2L17), CD4-BV421 (RPA-T4), CD25-BV510 (M-A251), CD69-PE (FN50), and CCR4-PE (L291H4) (Biolegend, San Diego, CA, USA). Analyses were performed with a FACS Canto II using FACS Diva (BD Biosciences), and FlowJo software (TreeStar, Inc., Ashland, OR, USA). Gating strategies for Tfh/Tfc and CRTh2^+^ T cells are demonstrated in Supplementary Figs. 1 and 2.

### 2.3 Allergen-specific T cell activation

PBMC (2 × 10^5^ per well) were incubated in U-bottom 96-well plates (Thermo Fisher Scientific, Waltham, MA USA) in 200 μl AIM-V medium (Thermo Fisher Scientific) as negative control, with 30 μg/ml PBS-extract from *Phleum pratense* pollen (Brehler et al., 2010) (Thermo Fischer Scientific, Allergon, Ängelholm, Sweden), Tetanus toxoid (TT) (Calbiochem San Diego, CA, USA) as antigen-specific positive control or with 1 μg/ml anti-CD3 monoclonal antibodies (mAb) OKT3 (Coulter, Miami, Fla., USA) maximum antigen independent stimulation. Allergen-reactive CD8^+^ T cells expressing CD25 were assessed by flow cytometry after incubation for 3 days at 37 °C as described [36].

### 2.4 Statistics

Statistical analyses were performed as indicated by using one-way ANOVA with Tukeys post-test, or student's t-test (GraphPad Software Inc., LaJolla, USA). Differences were considered significant if p ≤ 0.05.

## 3 Results

### 3.1 Circulating CD4^+^ Tfh cells during pre-seasonal allergoid AIT

**Fig. 2:**
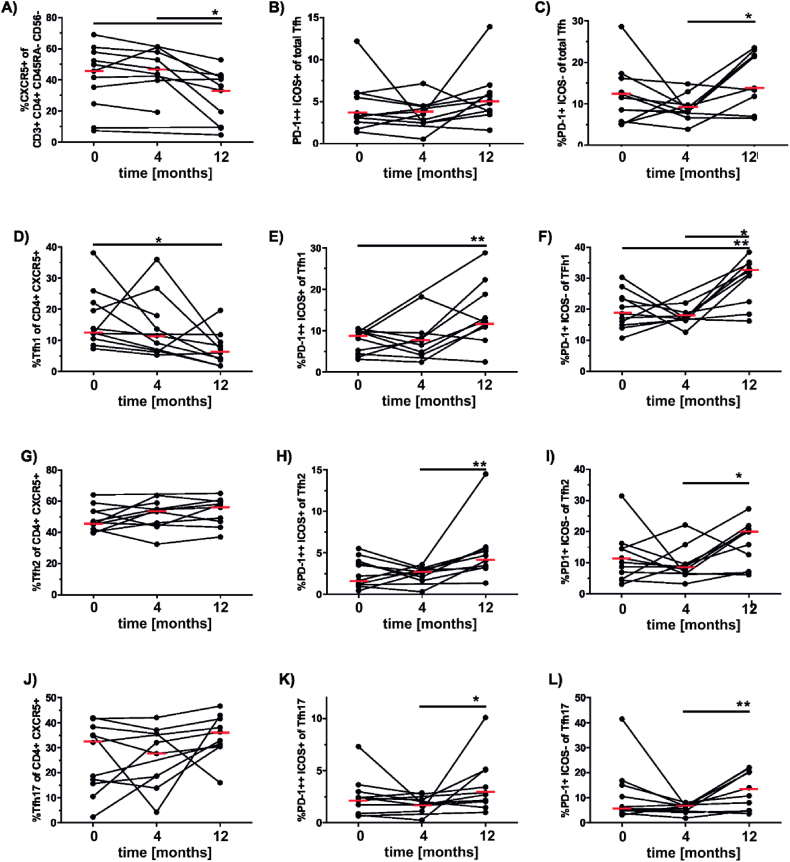
**Circulating Tfh subsets (CD4**^**+**^**follicular T cells) of the SCIT.** The percentages of different CXCR5^+^CD4^+^ T memory subsets in PBMC were assessed by flow cytometry. A. CXCR5^+^ CD4^+^ Tfh. B. and C. Activated Tfh expressing PD-1 or PD-1 and ICOS. D. CD4^+^ Tfh1 expressing CXCR3^+^. E. and F. Activated Tfh1 expressing PD-1 or PD-1 and ICOS. G. CD4^+^ Tfh2 negative for CXCR3 and CCR6. H. and I. Activated Tfh2 expressing PD-1 or PD-1 and ICOS. J. CD4^+^ Tfh17 expressing CCR6. K. and L. Activated Tfh17 expressing PD-1 or PD-1 and ICOS. Statistical significance was calculated by one-way ANOVA for repeated measurements followed by Tukeys multiple comparison post tests (*p < 0.05; **p < 0.01).

Potential changes of cTfh in 10 patients who developed a protective humoral response during allergoid AIT (schematically illustrated in Fig. 1 B),C), original data previously described in Ref. [37]) were monitored in samples collected at before, at 4 months, i.e. at the beginning of the maintenance phase, and at 12 months after the start of scAIT (Fig. 1). At the start of the therapy (t = 0), the percentages of total CXCR5^+^CD4^+^ Tfh within CD45RA^−^CD4^+^ memory T cells varied from 7.3 % to 69.0 % between the 10 individuals (Fig. 2A). Overall, the median of CXCR5^+^CD4^+^ Tfh did not change during the first 4 months of AIT. After 12 months of scAIT, most subjects showed a significant decrease in Tfh compared to the earlier time points (median: 34.7 %; Fig. 2A). The levels of activated PD-1^++^ICOS1^+^ Tfh (Fig. 2 B) (Schmitt 2014) increased marginally throughout the observed time period without reaching any statistical significance. Memory Tfh PD-1^+^ICOS^−^ displayed a slight decrease after the first 4 months the treatment, but significantly increased after 1 year compared to the 4 month timepoint (Fig. 2C).

Possible differential changes of the functionally different subsets of Tfh cells were investigated in Fig. 2 D-L. In general, before scAIT, the proportions of CXCR3^+^ Tfh1 cells (median: 12.4 %) were markedly lower than those of CCR6^+^ Tfh17 (32.2 %) or CXCR3^-^ CCR6^-^ Tfh2 cells (46.2 %). Over the course of the treatment the Tfh1 population gradually decreased and reached statistical significance after 12 months of treatment, compared to before (Fig. 2D). The activated Tfh1 subset did not change during the first 4 months, but significantly increased at 12 months (Fig. 2E). Similarly, the Tfh1 memory population did not change during the first 4 months and was significantly increased at the last observation point (Fig. 2F).

The Tfh2 subset displayed a slight increasing trend during the treatment period (Fig. 2G). The activated Tfh2 population also increased gradually over time reaching statistical significance at 12 months of therapy (Fig. 2H). Memory Tfh2 cells slightly decreased after 4 months of treatment and significantly increased after 12 compared to 4 months (Fig. 2I). The overall Tfh17 population did not change significantly in the course of treatment (Fig. 2J). The activated Tfh17 population displayed a significant increase at 12 months of treatment (Fig. 2K). The memory phenotype gradually increased over time with reaching statistical significance after 12 months compared to 4 months of treatment (Fig. 2 L).

### 3.2 Circulating CD8^+^ Tfc cells during pre-seasonal allergoid AIT

**Fig. 3:**
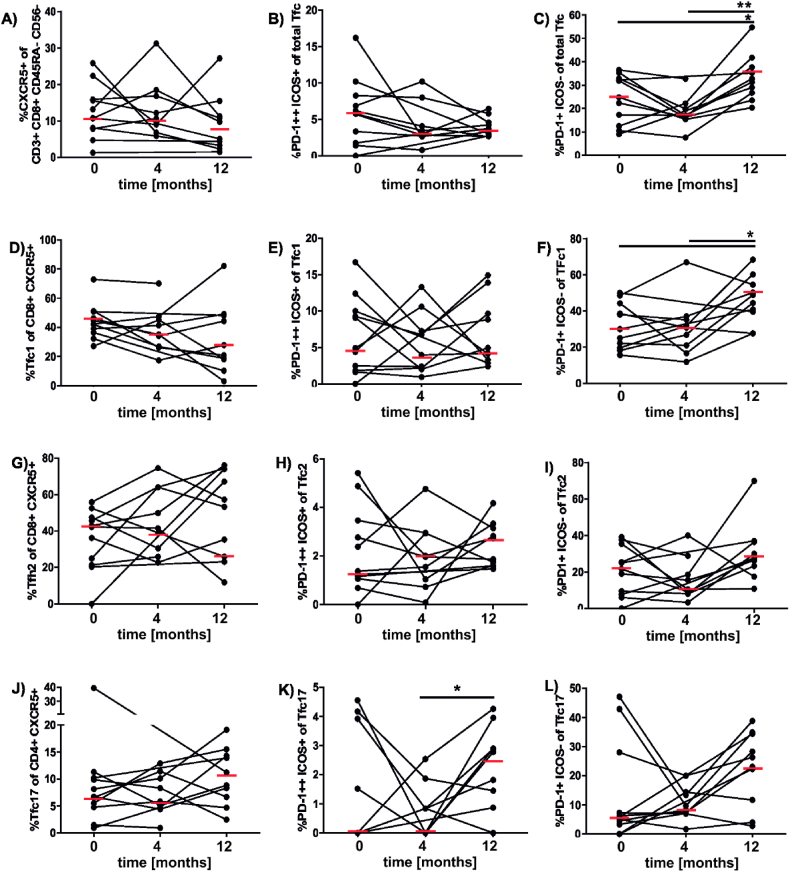
**Circulating subsets of CD8**^**+**^**follicular T cells (Tfc) during SCIT.** The percentages of different CXCR5^+^CD8^+^ T memory subsets in PBMC were assessed by flow cytometry. A. CXCR5^+^ CD8^+^ Tfc. B. and C. Activated Tfc expressing PD-1 or PD-1 and ICOS. D. Th1-like CD4^+^ Tfc expressing CXCR3^+^. E. and F. Activated Tfc1 expressing PD-1 or PD-1 and ICOS. G. Th2-like CD4^+^ Tfc negative for CXCR3 and CCR6. H. and I. Activated Tfc2 expressing PD-1 or PD-1 and ICOS. J. Th17-like CD4^+^ Tfc expressing CCR6. K. and L. Activated Tfc17 expressing PD-1 or PD-1 and ICOS. Statistical significance was calculated by one-way ANOVA for repeated measurements followed by Tukeys multiple comparison post tests (*p < 0.05; **p < 0.01).

Similar to Tfh cells, the percentages of Tfc showed high individual variability (Fig. 3). The fraction of CD45RA^−^CXCR5^+^ Tfc within the circulating CD45RA^−^CD8^+^ memory T cells was much smaller (median: 10.8 %) than the fraction of CXCR5^+^ Tfh in CD4^+^ memory T cells (median: 45.6 %; Fig. 3A). However, similar to the total Tfh population the Tfc population displays a trend to decrease over time. The percentages of activated PD-1^++^ICOS1^+^ Tfc before the treatment (median: 5.8 %; Fig. 3B) were similar to those of their CD4^+^ PD-1^++^ Tfh counterparts (median: 3,3 %; Fig. 2B). During treatment the levels of this population decreased slightly. Strikingly, the memory PD-1^++^ Tfc subset was significantly increased in PBMC that had been isolated after 12 months of scAIT when compared to the two previous timepoints (Fig. 3C). The subset distribution of Tfc was biased towards Tfc1 and Tfc2 which comprised 42.3 % and 42.2 %, respectively (Fig. 3D,G). Only 6.7 % of Tfc belonged to the Tfc17 subset (Fig. 3J). The activated Tfc1 subset did not show any changes during the analyzed time period (Fig. 3E). Interestingly, the memory subset was increased significantly at the last timepoint compared to the two previous (Fig. 3F). The percentages of total Tfc2 gradually decreased non-significantly during the therapy (Fig. 3G), while the percentages of activated Tfc2 increased non-significantly (Fig. 3H). The memory Tfc2 subset displays a small decrease after 4 months, but recovers again after 12 months (Fig. 3I). Total Tfc17 were found to be marginally increased after 12 months (Fig. 3J). Similarly, the activated Tfc17 population was unchanged after 4 months and increased after 12 months with statistical significance (Fig. 3K). The memory subset displayed a similar picture as the activated PD-1^++^ICOS1^+^ subset, however no statistical significance was observed (Fig. 3L).

### 3.3 CRTh2^+^CD4^+^ T cells and CRTh2^+^ CD8^+^ T cells during pre-seasonal allergoid AIT

**Fig. 4:**
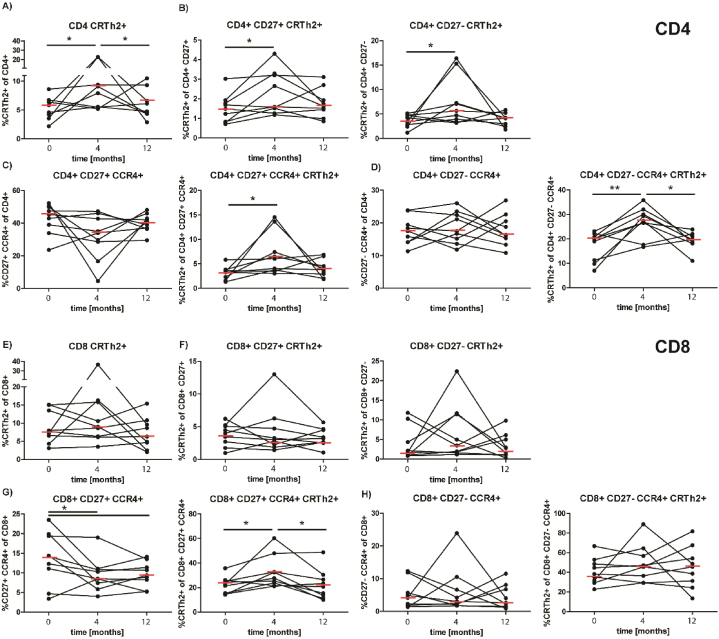
**Circulating CRTh2**^**+**^**CD4 and CRTh2**^**+**^**CD8 T cells during the course of SCIT.** The percentages of the different T cell subsets in PBMC were assessed by flow cytometry. A. CRTh2^+^ cells within CD4^+^ T cells. B. CRTh2^+^ positive cells within early (CD27^+^) and late memory (CD27^−^) CD4^+^ T cells. C. Early memory CD4^+^ T cells expressing CCR4 or CCR4 and CRTh2. D. Late memory CD4^+^ T cells expressing CCR4 or CCR4 CRTh2. E. CRTh2^+^ cells within CD8^+^ T cells. F. CRTh2^+^ cells within early and late memory CD8^+^ T cells. G. Early memory CD8^+^ T cells expressing CCR4 or CCR4 and CRTh2 H. Late memory CD8^+^ T cells expressing CCR4 or CCR4 and CRTh2. Statistical significance was calculated by one-way ANOVA for repeated measurements followed by Tukeys multiple comparison post tests (*p < 0.05; **p < 0.01).

The monitoring of total CRTh2^+^ and/or CCR4^+^ Th2 CD4^+^ T cells is shown in Fig. 4. After 4 months a significant increase of CD4^+^CRTh2^+^ T cells (Fig. 4 A) was observed, both, in early CD27^+^ as well as late CD27^−^ memory CD4^+^ T cells (Fig. 4 B). At 12 months the levels of early and late memory Th2 subsets had decreased to levels before scAIT. A similar initial increase was detected in CRTh2^+^CCR4^+^ double positive, early and late memory cells Th2 CD4^+^ T cells (Fig. 4C and D).

Within total CRTh2^+^CD8^+^ T cells, as well as in the early CD27^+^ and late CD27^−^ memory CRTh2^+^CD8^+^ T cells no significant changes were observed (Fig. 4E–H). Only, within CCR4^+^CD8^+^ T cells, the less differentiated CD27^+^CCR4^+^CD8^+^ T cells displayed a significant decrease after 4 month of treatment which persisted until 1 year. The CRTh2^+^ subpopulation was significantly increased after 4 months of treatment (Fig. 4 G), but recovered to baseline levels after 1 year of treatment. In the very late memory CD27^−^CCR4^+^CD8^+^ T cells no changes were observed in the total population. However, CRTH2^+^ shows a trend to increase during the observed time period.

**Fig. 5:**
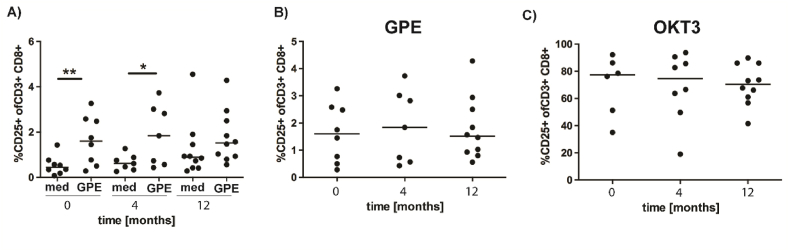
**Allergen-activated CD8**^**+**^**T cells during the course of SCIT treatment.** PBMC were stimulated with grass-pollen extract (GPE), or with anti-CD3 antibody (OKT3), or kept in medium (med) as negative control. After 3 days the percentages of activated CD25^+^ CD8^+^ T cells were determined by flow cytometry. A. Comparison of GPE-treated PBMC with medium control. B. Positive control, CD8^+^ T cell in PBMC cultures after stimulation with anti-CD3 (OKT3) antibodies. Statistical significance was calculated by paired t-tests in A) and one-way ANOVA for repeated measurements followed by Tukeys multiple comparison post tests in B) and C) (*p < 0.05; **p < 0.01).

In order to control that allergen-reactive CD8^+^ T cells were present, we applied an indirect test system described by Yu et al. [36]. PBMC were stimulated with grass pollen extract for 3 days and CD8^+^ T cells were analyzed for their expression of CD25. As positive control, PBMC were stimulated with OKT3. Medium alone served as negative control. As demonstrated in Fig. 5A, the percentages of CD25^+^CD8^+^ T cells were significantly higher in allergen-stimulated than non-stimulated cells at the onset and after 4 months, but not one year of scAIT. However, the percentages of CD25^+^CD8^+^ T cells in cultures stimulated with allergen or OKT3-did not differ significantly between the different timepoints (Fig. 5B and C).

## 4 Discussion

The effectiveness of allergoid AIT with extracts from airborne sources has been extensively described in mouse and human studies, but the measures in these studies were mostly the clinical improvement or the anticipated protective antibody response [[38], [39], [40], [41], [42]]. However, around the millennium Fiebig et al. stimulated PBMC of allergic donors with grass pollen allergoids to obtain allergen-reactive T cell lines [43,44]. Furthermore, one study demonstrated increased proliferative responses of antigen-specific immune cells, including T cells, before the end of a 4-week AIT regimen [45]. In our study grass pollen allergy was treated with increasing doses of vaccine up to the maintenance dose followed by natural pollen exposure. As these patients undergoing this pre-seasonal allergoid AIT showed marked allergen-specific humoral responses, we sought to analyze the responses of different T cell subsets during their first year of treatment.

Different subsets of follicular helper T cells were monitored. Tfh cells have been recently described as major players in the promotion of antibody isotype switching towards IgE [9]. As generally an immune deviation from Th2 to Th1 has been supected in AIT [46,47], also functional changes within Tfh have been suspected. Only limited data on Tfh cells during the course of AIT have been reported, especially because of their low abundance and difficulty of detection. In one study of oral AIT, where patients receive daily high doses of allergen, allergen-specific Tfh cells could successfully be monitored and a role in immunotherapy was suggested [48].

Due to the multiple weekly allergen injections that induced humoral responses [37], we expected changes at the level of Tfh cells already at 4 months of scAIT. Interestingly, a sequential significant decrease of total Tfh cells was observed throughout the treatment period. A similar trend decrease was seen in the total Tfc population. This observation can possibly be due to the different sizes of those population. Whereas the Tfh account for roughly 45 % of the CD4 population (Fig. 2A), the Tfc only account for around 10 % of the CD8 population (Fig. 3A). Hence we assume that the Tfc, despite their similar characteristics ([49]), may play a smaller role compared to their Tfh counterparts.

After dissecting these populations into their functionally different subsets we identified no change in the Tfh1 population at 4 months of treatment. In the Tfh2 subset we observed an early trend to increase, whereas a marginal decline in the Tfh17 subset was revealed. This finding was in line with previous reports by others on enhanced proliferative T cell responses and elevated levels of Th2 cytokines during early scAIT, supposedly reflecting the stimulation of already pre-existing Th2 cells [4,50]. To increase the sensitivity of T cell phenotyping by analyzing special subsets we assessed the amounts of activated of Tfh and Tfc cells by staining for PD1 and ICOS expression. However, *in toto* the percentages of activated circulating cells did not change after 4 months of treatment in our study cohort (Fig. 2, Fig. 3).

After twelve months of scAIT the percentages of total Tfh cells were significantly decreased compared to pre-treatment (Fig. 2, Fig. 3A). For the total Tfc population a similar trend was observed. This finding was in line with previous studies. Schulten et al. had shown that the percentage of total Tfh cells decreased in the course of at least of 6 months of AIT [21]. This decrease has been described -also by others-to be accompanied by an increase of regulatory Tr1 cells [4,21]. Interestingly, all Tfh subsets showed significantly higher percentages of activation at the 12-month time point compared to the 4-month time point. The same trend was found for Tfc cells. We hypothesize that this phenomenon may result from recent seasonal viral infections which occur more frequently during winter as compared to the 4-month time point at the end of spring [11,51]. In line, the Tfh1 and Tfc1 populations showed a significantly higher activation at 12 months compared to before the treatment possibly indicating an increased viral challenge during the winter months compared to the year before (Fig. 2, Fig. 3F). The second possibility for this observed phenomenon could be the compensation of the lower cell number by an higher activation status.

Aside from Tfh cells we also addressed changes in so-called allergenic T cells (Fig. 4). This recently described T cell subset is characterized by surface expression of CRTh2 [52]. Most interestingly we found allergenic CRTh2^+^ T cells increased early after 4 of treatment, not only significant increases in percentages, but also in the state of activation within the CD4^+^CRTh2^+^ population. After 1 year the levels were back at baseline levels. In contrast, *Wambre* and colleagues observed a substantial decrease of this subset after 1 year [52]. These changes were in line with the treatment scheme, as the patients were administered with increasing doses of the allergens during this period. However, after withdrawal of the treatment and only seasonal exposure, the changes were reverted. A possible explanation could be the exposure to relatively low amounts of allergens during the pollen season, leading to no signs of exhaustion in this subset. Regarding measurable Th2 responses in PBMC, a strong dependence on the extent of pollen exposure has been previously observed in mugwort pollen allergy [53].

The CD8^+^CRTh2^+^ T cells showed similar, but less prominent changes than their CD4^+^ counterparts. Interestingly, the CCR4^+^CRTh2^+^ subset was significantly increased after 4 months of treatment, opposed to the total CRTh2^+^ population. This effect may reflect the early Th2 response elicited by the immunotherapy, as CCR4^+^ T cells as well as IL-4 production are boosted during allergic inflammation [54]. Thus, the obtained data indicate that the seasonal exposure after pre-seasonal vaccinations is not sufficient to induce sustained, detectable changes on T cell levels, when compared to conventional AIT with continuous administration of allergens.

Together, in contrast to the significant changes observed for the allergen-specific humoral responses reported during pre-seasonal scAIT [37], changes at the T cell level were far less prominent in these patients. The little difference among the timepoint can be attributed to various factors. The small patient cohort and the high individual variability are accountable. Additionally, the study was solely observational in a selective population without a placebo cohort and could not be compared against healthy individuals. Nonetheless, the observed data are in line with previously reported data from allergoid AIT treated patients [1,40,41]. We added to this existing knowledge on allergoid AIT, by studying the accompanying T cell changes during the course of a pre-seasonal allergoid AIT. Interestingly, also the adjuvant MPL has been described to first induce an early activation of T cells similar as in our study and only later an induction of Treg cells starting after 2 years of treatment [42,45]. The analysis of allergenic CRTh2^+^ T cells demonstrated that the immunological changes during pre-seasonal treatment of pollen allergy depend to a high extent on the booster effect during the grass pollen season. Therefore, the classical administration scheme with continuous monthly allergen injections during the maintenance phase of scAIT may induce larger immunological changes, as the comparison of humoral changes in the studied subjects has suggested previously ([35,42]).

## Author contribution statement

M.R.; C.K.; B.B.; B.J.S. conceived and designed the experiments.

M.R. performed the experiments.

M.R.; C.K., B.B.; B.J.S. analyzed and interpreted the data.

S.L.B.; F.H. contributed reagents, materials, analysis tools or data.

M.R.; B.B, B.J.S. wrote the paper.

## Data availability statement

Data will be made available on request.

## Declaration of competing interest

All authors declare to have no commercial or financial conflict of interest.
