# Supplementary material for: T-cell subset changes during the first year of pre-seasonal allergoid allergen-specific immunotherapy
Source: Heliyon. 2023 Nov 10;9(11):e21878. doi: 10.1016/j.heliyon.2023.e21878 (PMC10685201; doi:10.1016/j.heliyon.2023.e21878)
Supplement: Multimedia component 1 [file mmc1.docx]

Supplement tables and figures

|  |  |  |  |  |  |  | specific IgE (kIU/L) | |
| --- | --- | --- | --- | --- | --- | --- | --- | --- |
| Patient | Age | Sex | Sensitization* | Manifestation** | total IgE (kU/L) | Phl p | Bet v | Der p |
| 1 | 19 | M | gp, bi | RC | 827,0 | >100 | 54,7 |  |
| 2 | 30 | M | gp | RC | 46,7 | 13,2 |  |  |
| 3 | 43 | M | gp, bi | RC | 151,0 | 2,2 | 12,8 |  |
| 4 | 32 | M | gp | RC | 62,0 | 3,5 |  |  |
| 5 | 21 | F | gp, bi | RC | 446,0 | 26,2 | 4,6 |  |
| 6 | 27 | M | gp, bi | RC , Aab | 209,0 | 22,5 | 57,0 |  |
| 7 | 36 | M | gp, bi, hdm | RC | 64,7 | 8,2 | 4,3 | 2,9 |
| 8 | 33 | F | gp,hdm | RC | 52,6 | 5,47 |  | 5,4 |
| 9 | 33 | F | gp | RC | 169 | 11,5 |  |  |
| 10 | 28 | M | gp | RC | 561,0 | 63,7 |  |  |

**Table S1: Characteristics of the study population (adapted from Reithofer et al. 2018)**

* gp, grass pollen; bi, birch pollen; hdm, house dust mite; **RC, rhinoconjunctivitis, Aab, allergic asthma bronchiale; Patients 1, 3 and 6 got SIT with Allergovit against grass pollen and birch pollen in parallel; Total serum IgE and GP-specific IgE was determined by ImmunoCAP (Thermo Fischer Scientific, Phadia AB, Uppsala, Sweden)


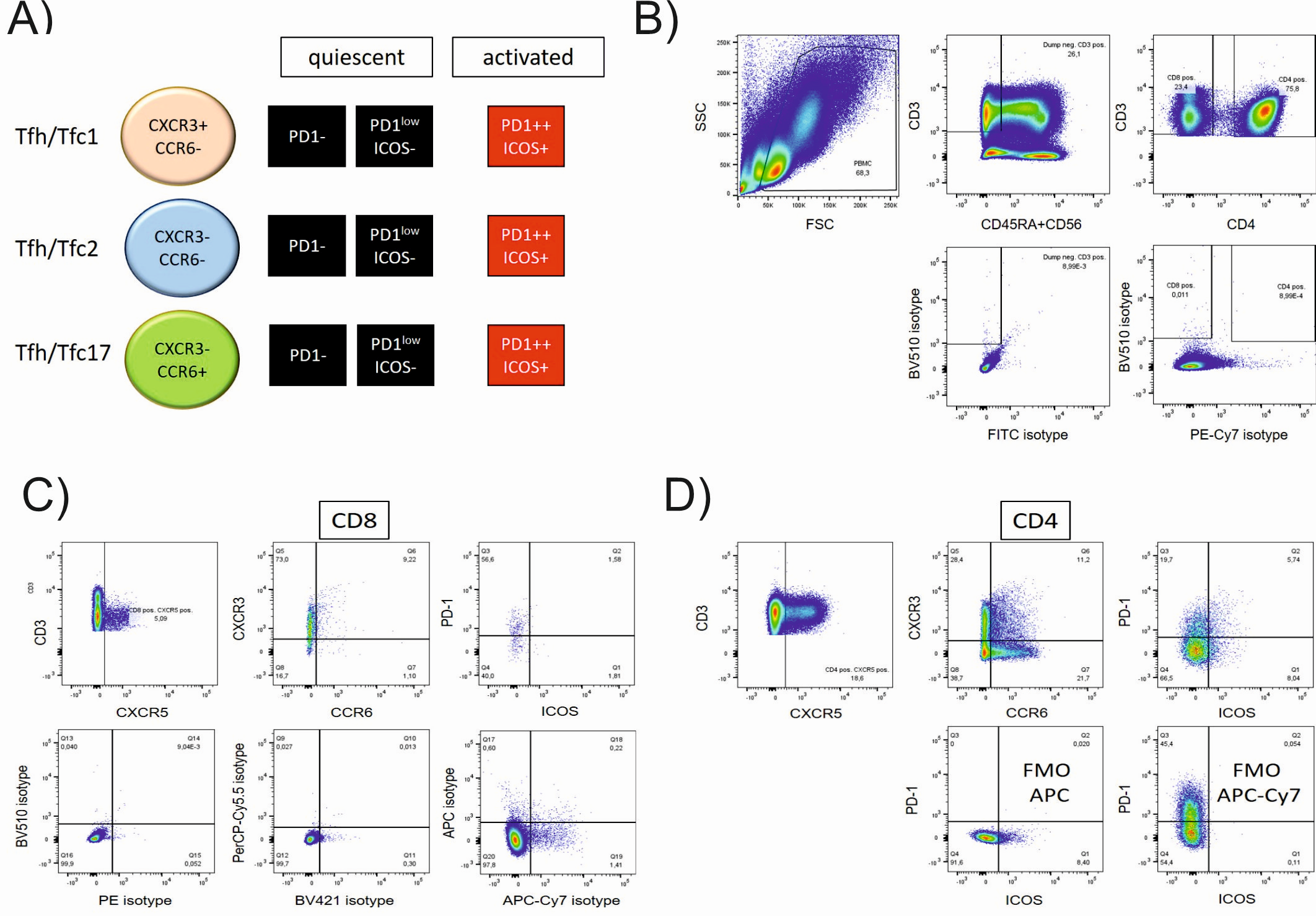


**Suppl. Figure 1: Gating strategy for Tfh/Tfc.** Flow cytometry data of a representative patient are shown. A. depicts the analyzed cell types. B. Represents the first gating steps leading to the CD8 and CD4 population. The respective isotypes are shown below. C. Gating for CD8 Tfc discrimination and activation determination is shown. Below the respective isotype controls. D. Gating for CD4 Tfh discrimination and activation determination is shown. Below the respective FMO controls for the activation markers. All isotype and FMO plots derive directly from the PBMC gate, without further modification.


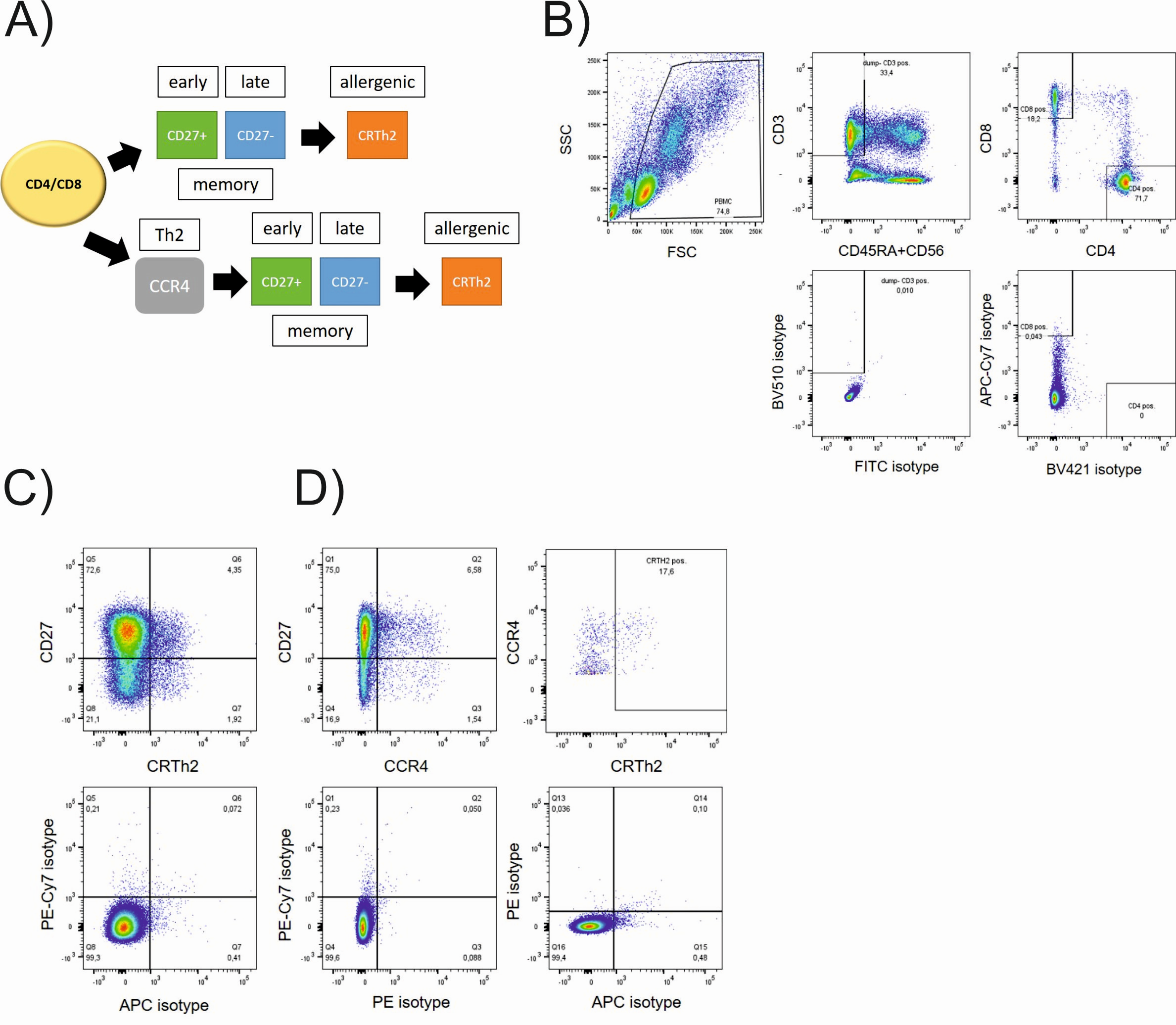


**Suppl. Figure 2: Gating strategy for CRTh2^+^ T cells.** Flow cytometry data of a representative patient. A. Schematic illustration of cell types analyzed. B. After gating live cells, CD3^+^ T cells were selected and subdivided into a CD4^+^ and a CD8^+^ T subset. Respective isotype controls are shown below. Within these two populations further assessed for their expression of CD27/CRTh2 C. or CD27/CCR4 in D. CCR4 and CRTh2 double positive cells were gated from CCR4^+^ cells. Respective isotype controls are shown below. All isotype and FMO plots derive directly from the PBMC gate, without further modification.


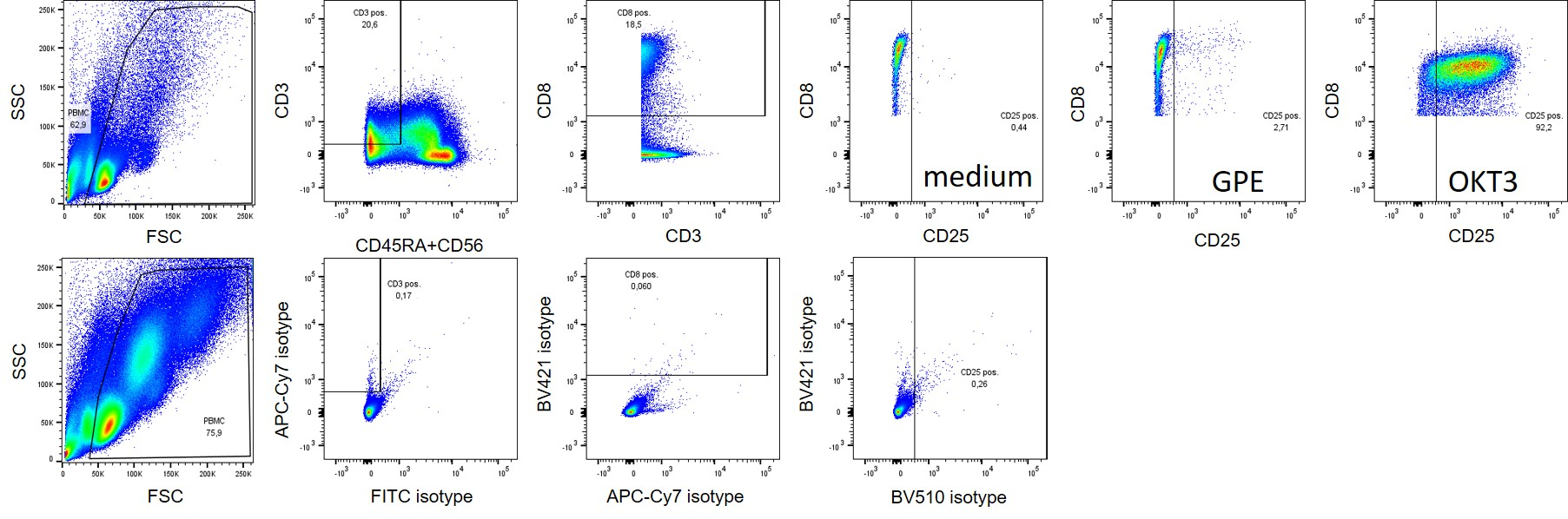


**Suppl. Figure 3: Gating strategy for allergen-activated CD8+ T cells.** Flow cytometry data of a representative patient. After gating live cells, CD3^+^ and CD8^+^ T cells were selected. The activation was assessed by gating for CD25 expression. Respective isotype controls are shown below. All isotype and FMO plots derive directly from the PBMC gate, without further modification.


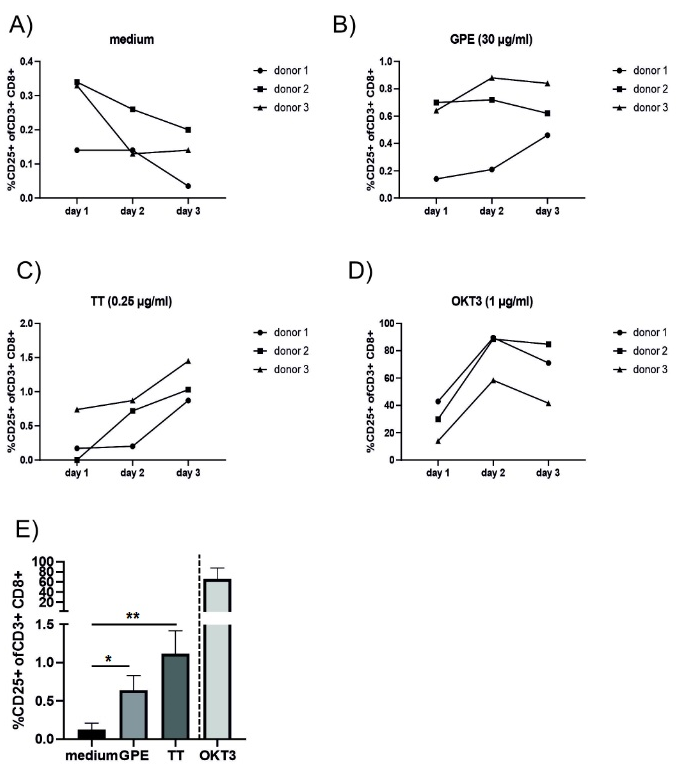


**Suppl. Figure 4: Establishment of antigen-specific CD8+ T cells stimulation.** A.-D. depicts the time course of CD25 expression of 3 different donors upon stimulation as indicated. E. Summary of the single stimulations. Each group consists of the 3 donors shown above at day 3 of stimulation. TT served as positive control for antigen-specific stimulation. OKT3 stimulation shows the full potential of antigen independent stimulation of CD8+ T cells among those patients. Statistical significance was calculated by one-way ANOVA for repeated measurements followed by Tukeys multiple comparison post tests for the antigen-specific stimulations with medium, GPE or TT stimulations (* p < 0.05; ** p < 0.01).
